# Effect of ZnO on Properties of Gels for Heritage Objects Conservation

**DOI:** 10.3390/gels7040251

**Published:** 2021-12-07

**Authors:** Oana-Cătălina Mocioiu, Irina Atkinson, Ana-Maria Mocioiu, Simona Neagu, Robert Ruginescu, Raul-Augustin Mitran, Mădălin Enache

**Affiliations:** 1 Institute of Physical Chemistry Ilie Murgulescu of the Romanian Academy—ICF, 202 Splaiul Independenţei, 060021 Bucharest, Romania; irinaatkinson@yahoo.com (I.A.); raul.mitran@gmail.com (R.-A.M.); 2 National Research & Development Institute for Non-Ferrous and Rare Metals—IMNR, 102 Biruinţei Blvd., 077145 Pantelimon, Romania; 3 Institute of Biology Bucharest of Romanian Academy—IBB, 296 Splaiul Independenţei, P.O. Box 56–53, 060031 Bucharest, Romania; simona.neagu@ibiol.ro (S.N.); robert.ruginescu@ibiol.ro (R.R.); madalin.enache@ibiol.ro (M.E.)

**Keywords:** SiO_2_-ZnO gels, historical glasses, sol–gel method, FTIR, antibacterial tests

## Abstract

One of the current research objectives is the development of new films for the conservation of glass heritage objects. The value of historical glass objects is given by the technology and raw materials used in production as well as their transparency and color. Their colors are correlated with oxide composition rich in transitional metals, which decrease resistance of corrosive agents from the atmosphere. In this paper, SiO_2_-ZnO gels have been designed to protect historical glass objects. The sol–gel method used to obtain gels is a powerful tool for functionalizing different materials. An important functionalization is the antibacterial activity. By applying a gel, the coated material is able to decrease the growth of bacteria. After deposition, some gels must be strengthened by heat treatment. The effect of ZnO content (10 mol% and 20 mol%) on the properties of the studied gels was investigated by Differential scanning calorimetry (DSC), Fourier transform infrared (FTIR), X-ray diffraction (XRD), Scanning electron microscopy (SEM), and antibacterial tests. *Escherichia coli* ATCC 25922, *Staphylococcus aureus* ATCC 25923, and the halotolerant bacterium, *Virgibacillus halodenitrificans*, isolated from a salt crystal from Unirea mine, Slănic Prahova, Romania, were used. The gel Gel 2 (SiO_2_-ZnO (20 mol%)) showed the best properties.

## 1. Introduction

Heritage objects from glass such as windows [1], stained glass windows [2,3], pottery [4], and mosaics [5,6] can be protected from the destructive activity of bacteria and environmental acid agents by utilizing organic or inorganic coatings. The materials used for coatings must meet conditions such as high transparency and antibacterial properties. Glass was widely used to embellish churches and cathedrals with colorful window panels, but it is often subjected to degradation due to its interaction with the ambient atmosphere, especially in the last few centuries [1]. The medieval stained-glass window is essentially a mosaic of colored pieces, each color being separated by lead ‘‘calms”, (strips made with cast lead) [3]. The concentration of silica in the glass usually determines its stability, and in the Monastery of Batalha its concentration is low enough to predict an unstable glass [3]. Early stained-glass artists were limited to a very few colors (blue, brown, green, red, violet, yellow) produced by adding oxides of d transition elements, such as Fe, Mn, Cu, etc., in the melting pot, where they were fused with the glass [3]. The most of the historical windows are since Roman and Medieval period. The composition of Roman glass is also compared with that of some Medieval and 18th-century glass [2]. The glasses differ in the type of flux, in that Roman and high medieval glass, natron, and late Medieval and modern glass plant ash, soda, and potash, respectively, were used [2].

It is known from the specialized literature that the historical objects were produced from soda-lime-silicate and lead silicate glasses. Historic glass objects can be preserved by coating with silica gels [7,8,9]. Sometimes they are heat-treated at temperatures below 500 °C, the temperature corresponding to the softening point of the glass. In the case of glasses with a composition of 70 wt% PbO and 30 wt% SiO_2_, similar with Japanese ones, the melting temperature was 850 °C [10]. Soda-lime glasses have a melting temperature higher by 200–400 °C. Due to the different softening and melting temperatures of the glasses, the working method and the composition of the protective gels must be adapted. To obtain antibacterial properties, oxides such as ZnO, CaO, MgO or Ag_2_O are added to the composition of the gels [11,12,13,14,15,16]. Many papers have reported antibacterial properties of ZnO-containing nanomaterials [12,13,14,15,16,17,18,19]. Tatar [20] reported obtaining of antibacterial transparent thin films containing different amounts of silver ion on the glass substrate. Thin films were obtained from inorganic-organic hybrid sols derived from 3-(glicydiloxypropyl) trimethoxy silane, N-(2-aminoethyl)-3-aminopropyl- trimethoxysilane, and aluminum-sec-butoxide/ethylaceto-acetate complex and doped with silver ions. The antibacterial activity of the coatings was investigated against Gram-negative *Escherichia coli* and Gram-positive *Staphylococcus aureus*. The results reveal that 1% doping of the transparent hybrid thin film with silver ions resulted in high antibacterial properties [20].

In the previous work of the authors [21] thin films of silica doped with 3 mol% and 5 mol% ZnO were studied and tested against acid corrosion and *Escherichia coli* effect. After 24 h can be seen bacterial reduction. SiO_2_-based materials doped with 1%, 3%, and 5% ZnO obtained by the sol–gel method [7] were characterized after thermal treatment at 700 °C by XRD measurements and FTIR spectroscopy and the non-crystalline structure was determined.

In this work, the effect of ZnO on the antibacterial properties and transparency of SiO_2_-ZnO gels obtained by sol–gel method and heat-treated at 400 °C were investigated. It was evaluated the possible growth-inhibitory effect over a wide concentration range (0.05–2 mg/mL) on reference Gram-negative and Gram-positive bacterial strains, *Escherichia coli* ATCC 25922 and *Staphylococcus aureus* ATCC 25923. Along with these relevant model microorganisms, *Virgibacillus halodenitrificans*, isolated from the natural environment from Romania [22,23], was also used for the studies.

## 2. Results and Discussion

In the system SiO_2_-ZnO were prepared, three gels noted S (SiO_2_) as a reference, G1 (SiO_2_-ZnO (10 mol%)) and G2 (SiO_2_-ZnO (20 mol%)).

### 2.1. Differential Scanning Calorimetry (DSC)

**Figure 1:**
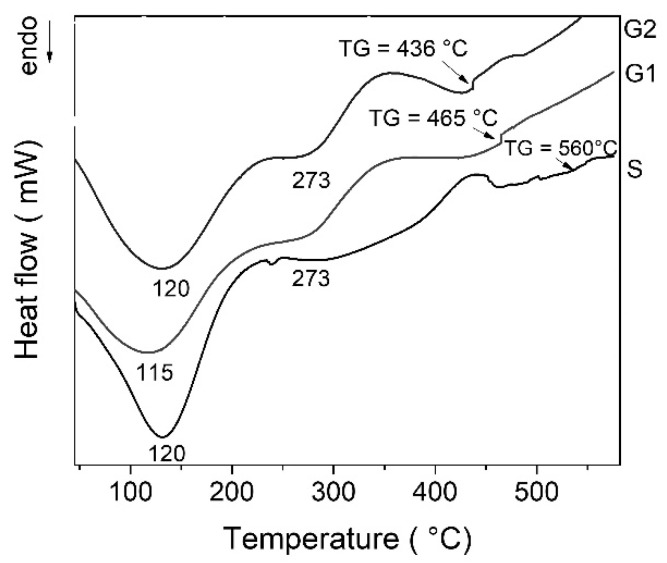
DSC curves of the gels.

The DSC curves of all gels exhibit multiple thermal effects (Figure 1). The first endothermic effect with a maximum at ~120 °C can be attributed to physisorbed water desorption. The decomposition of residual organic could be responsible for the exothermic effect around 350 °C [24]. The characteristic glass transition (TG) can be noticed for each gel at temperatures above 430 °C. Increasing zinc oxide content leads to decrease of temperature for water and organics elimination and also decreased glass transition temperature (TG). After deposition, some gels must be strengthened by heat treatment. In this case, the treatment temperature must be set below the glass transition temperature of the glass object. Considering thermal behavior of the gels, thermal treatment was established at 400 °C for 3 h.

### 2.2. X-ray Diffraction (XRD)

**Figure 2:**
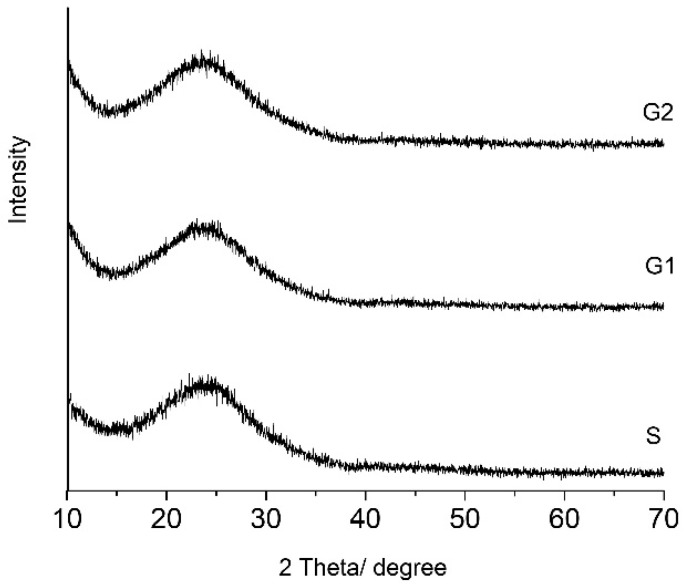
XRD patterns of the gels thermally treated at 400 °C.

All gels remained amorphous even after thermal treatment at 400 °C for 3 h they as can be seen in the Figure 2. All gels have more than 80 mol% SiO_2_ in composition. The broad peak at 2θ = 15 to 30 was attributed to silica gel [25]. There are no differences between the shapes of pattern in the gels. Zinc oxide can act in vitreous systems as a former or a modifier of the network. In the studied hybrid gels, zinc oxide plays the role of a modifier. Up to 20 mol%, the zinc oxide did not act as a former and did not make its own network.

### 2.3. Fourier Transform Infrared (FTIR)

**Figure 3:**
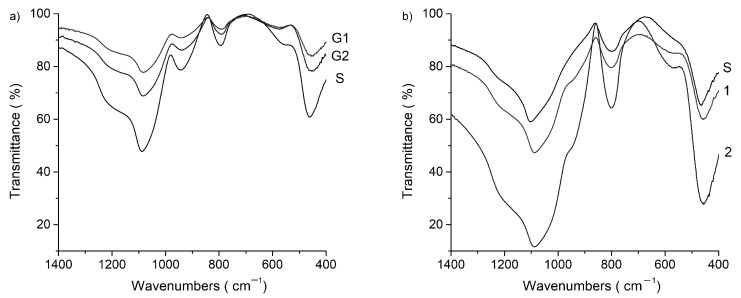
FTIR spectra in the range 400–1400 cm^−1^ for gels (**a**) dried and (**b**) treated at 400 °C.

Table 1 shows the position of the main characteristic bands in the FTIR spectra in the 400–4000 cm^−1^ domain. Figure 3 exhibit the range of interest for Si–O and Zn–O bonds, between 400 cm^−1^ and 1400 cm^−1^. The shape of all spectra is characteristic to amorphous state as can be seen from the presence of wide bands. Infrared spectra of all the gels display four or five wide bands within 400–1400 cm^−1^ region specific to disordered silicate network. According to the literature data [19] bands near 1220 and 1088 cm^−1^ are assigned to asymmetric stretching modes of the Si–O–Si for various [SiO_4_] tetrahedra with n bridging oxygen atoms (BOs), Qn units, while band at 945 cm^−1^ corresponds to non- bridging oxygen Si–O stretching modes (Si–O–NBO). The presence of Q3 units therefore shows a higher polymerization.

Other important information given by the band intensities are related to the transparency of the gels. In the spectra of gels dried at 100 °C transmittance is high (over 80%) and this can be correlated with good transparence of gels. In the spectra of gels heat-treated at 400 °C, the bands intensities increase, transmittance decrease until 60%, and gel transparence also. The intensity of the absorption bands increases after heat treatment and with ZnO concentration.

In the S (SiO_2_) gel spectrum, the main bands are wide due to the amorphous state. The most intense between 1400 and 980 cm^−1^ peaking down at 1088 cm^−1^ has one shoulder at 1209 cm^−1^. The bands of 1088 cm^−1^, 795 cm^−1^ and 460 cm^−1^ are characteristic of the structural units [SiO_4_] tetrahedra linked together. The spectra show the peak associated with the TO vibration mode of the asymmetric stretch of Si–O–Si bonds located at 1088 cm^−1^, and the shoulder at approximately 1209 cm^−1^ is assigned to the LO vibration mode of Si–O–Si. The temperature led to ordering of the structural units of silica. In addition, bands at 945 cm^−1^ that are attributed to Q1 (pair of tetrahedra), it turns into a shoulder after treatment at 400 °C. Additionally, the band at 540 cm^–1^ disappears after heat-treatment.

In the gels with zinc oxide in composition the bands intensities increase more after heat-treatment and the bands at 945 cm^−1^ turns also into a shoulder. The Zn–O–Zn stretching pattern is observed at almost 575 cm^−1^, and the intensity of this band increases with the ZnO content. The absorption bands at 460 cm^−1^ in the silica gel spectrum are shifted to smaller wavenumbers in ZnO-containing gels, which could be attributed to the formation of the Zn–O–Si bond. The intensity of this band increases after treatment at 400 °C.

### 2.4. Scanning Electron Microscopy (SEM)

**Figure 4:**
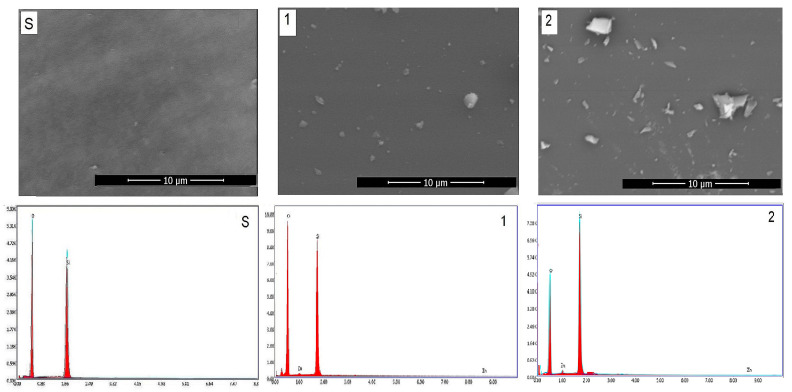
SEM and EDAX results.

**Figure 5:**
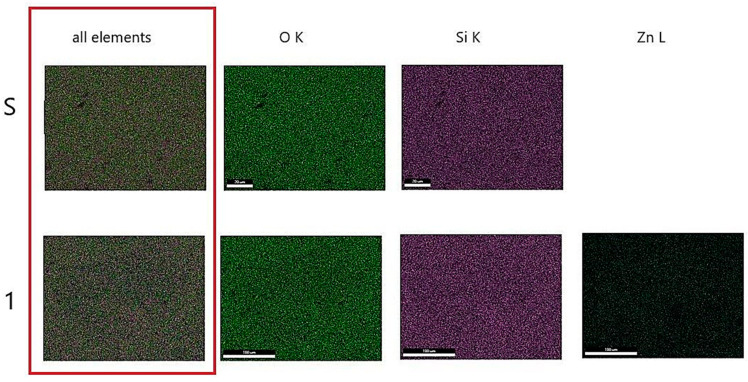
SEM mapping of the treated gels.

Figure 4 shows the SEM/EDAX of gels obtained by the sol–gel method and those heat-treated at 400 °C for 3 h. SEM images show the typical morphologies of vitreous materials in the form of matrix (gray background) and white crystallites. The crystallite dimensions were below the detection limit of the XRD apparatus. EDAX results on the surface reflects the chemical composition. The number and size of crystallites increase with the content of zinc oxide. Figure 5 shows the mapping for heat-treated gels at 400 °C called S (SiO_2_) and 1 (10 mol% ZnO). The figure shows the uniform distribution of zinc ions on the surface of Gel 1 (10 mol% ZnO) heat-treated at 400 °C.

### 2.5. Antibacterial Tests

**Figure 6:**
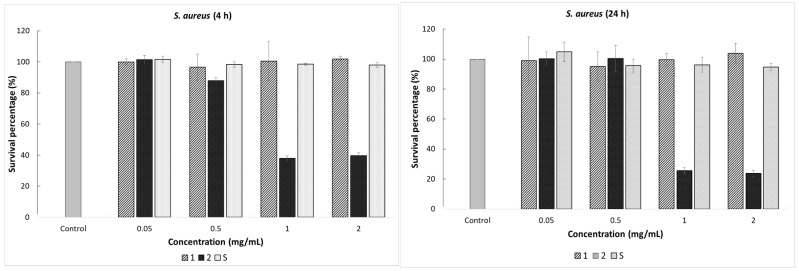

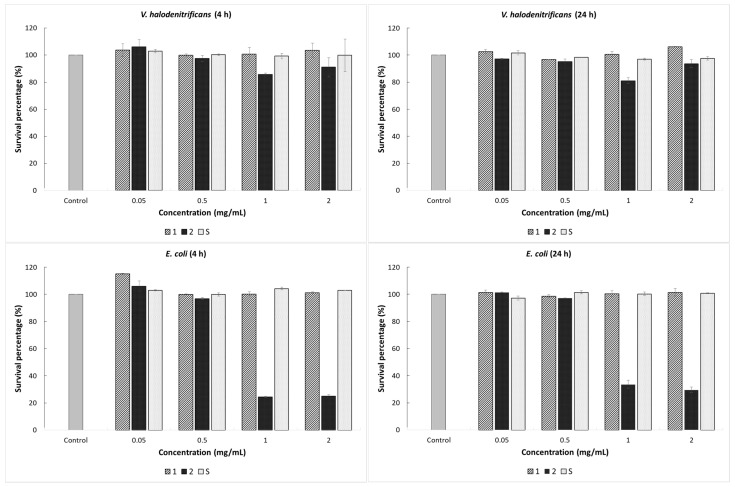
The survival percentage of the bacteria after exposure to 0.05, 0.5, 1, and 2 mg/mL of synthesized gels after thermal treatment (1, 2, S), after 4 and 24 h of incubation in liquid culture medium specific to each bacterium, compared with the control untreated cells. The columns in the histogram represent the mean ± standard deviation of two replicates.

The antimicrobial assay was performed against *E. coli* ATCC 25922, *S. aureus* ATCC 25923, and *V. halodenitrificans*, a halotolerant bacterium isolated from a rock salt crystal. The bacterial strains were selected to represent both Gram-positive and Gram-negative bacteria and observe, by comparison, the response of the cell to the action of the SiO_2_-ZnO gels after treatment at 400 °C for 3 h. Viability of the bacterial cells with and without the addition of (0.05, 0.5, 1, and 2 mg/mL) synthesized gels was evaluated, and the results are shown in Figure 6. Data on the percent survival of the bacterial strains after exposure to the materials tested in the concentrations of 0.05 and 0.5 mg/mL, respectively, revealed no significant change in cell viability. Further, we found that the treatment with 1 and 2 mg/mL of vitreous counting caused a different bacterial cell response. Therefore, the addition of Gel 2 (SiO_2_-ZnO (20 mol%)) treated at 400 °C generated a significant effect on the cells of *S. aureu*s and *E. coli*, reflected by the decrease in microbial population density.

As shown in Figure 6, the lowest percentage of survival bacterial was found at 24 h of incubation and 2 mg/mL amount of material, with the value of 23.5% for *S. aureus* in comparison with the control sample. According to the obtained results, the impact of Gel 2 (SiO_2_-ZnO (20 mol%)) after treatment at 400 °C on the *E. coli* bacterial cells was much higher in the first incubation hours. Thus, for material concentrations of 1 and 2 mg/mL, the survival percentages after 4 h of treatment were 24.56% and 25.17%, respectively. The average microbial population has slightly increased by applying a longer exposure time, with the values of 33.73% and 29.66%, respectively. This initial decrease of the cell viability could be due to material stress on the growth of the bacterium, whereas the relative increase afterward is consistent with bacterial adaptation to the presence of the gel. The main finding reported here is that the inhibitory effect of the synthesized gels is due to the synergistic effect between SiO_2_ and ZnO and the quantity of synthesized gels.

Gel 1 (SiO_2_-ZnO (10 mol%)) and gel S (SiO_2_) after treatment at 400 °C in the tested concentrations showed no antibacterial activity on the *V. halodenitrificans*. Only Gel 2 exhibited a low inhibitory effect on this strain. In the case of Gel 2, (SiO_2_-ZnO (20 mol%)) after 24 h of contact, the bacterial reduction was 18.8%; and the cell density decreased from 6.4 × 10^14^ CFU/mL to 5.2 × 10^14^ CFU/mL. Here, the time factor does not seem to influence the antibacterial capacity of the composite material. Thus, no significant decrease in the bacterial outgrowth was recorded even after 48 h of treatment. Moreover, this resistance of the bacterium to the action of synthesized gels can be explained by the presence of large amounts of exopolysaccharides that halophilic microorganisms can synthesize, thus representing a protective mechanism. The fact that gels exerted a lower impact on *V. halodenitrificans* than on the other two tested microorganisms, under the same conditions, indicates that the response of bacteria to gels is species-dependent.

## 3. Conclusions

In the SiO_2_-ZnO system, gels with 10 mol% ZnO and 20 mol% ZnO were obtained by sol–gel method. The ZnO content induced differences in the structure and antibacterial properties of the gels.

Differential scanning calorimetry (DSC) evidenced the removal of water and organic compounds up to 400 °C; and glass transition temperature (TG) above 430 °C correlated with zinc oxide content.

The FTIR spectra confirm the vitreous state of SiO_2_-ZnO gels. In the spectra of gels dried at 100 °C transmittance is high (over 80%) and this can be correlated with good transparence of gels. In the spectra of gels heat-treated at 400 °C, the bands intensities increase, transmittance decrease until 60%, and gel transparence also, but is still in the field of uses. The intensity of the absorption bands increases after heat treatment and with ZnO concentration.

Antibacterial tests showed for Gel 2 (SiO_2_-ZnO (20 mol%)) the presence of an inhibitory effect on the reference bacterial strains. Gel 1(SiO_2_-ZnO (10 mol%)) and gel S (SiO_2_) act as bacteriostatic agents.

## 4. Materials and Methods

### 4.1. Sol–Gel Method

The solutions have been prepared using TEOS (tetraethyl orthosilicate) [Si(OC_2_H_5_)_4_], and zinc acetate dehydrated as sol–gel precursors, ethanol as solvent, distilled water for hydrolysis, and HCl as a catalyst. The equivalent molar ratio of each was: C_2_H_6_O:TEOS:H_2_O:HCl = 10:1:3:0.03. After 30 min of stirring, the zinc acetate dehydrated in the desired amount was added to each solution. After aging for 24 h, obtained gels were dried at room temperature, in air, and then they were annealed at 400 °C for 3 h. The dried gels were noted S (silica gel), G1 (with 10 mol % ZnO), and G2 (with 20 mol % ZnO). All reageants used in this section were purchased from Sigma-Aldrich, Saint Louis, MI, USA.

### 4.2. Gels Characterization

Differential scanning calorimetry (DSC) analyses of gels were performed using a Mettler–Toledo DSC 3 calorimeter (Thermo Fisher Scientific Inc., Waltham, MA, USA), at a heating rate of 10 °C/minute, under a nitrogen atmosphere and in the temperature range of 20 °C to 600 °C. Based on DSC results, gels were thermally treated at 400 °C time to 3 h.

X-ray diffraction (XRD) of the gels was performed using a Rigaku Ultima IV diffractometer (Rigaku Corporation, Tokyo, Japan) in parallel beam geometry equipped with CuKα radiation (wavelength 1.5406 Å) in 2θ range between 10 to 70 with a speed of 2°/min and a step size of 0.02°.

Fourier transform infrared (FTIR) spectra of the gels were recorded with a Nicolet 6700 (Thermo Fisher Scientific Inc., Waltham, MA, USA) apparatus in 400–4000 cm^−1^ domain, with a sensibility of 4 cm^−1^. The finely ground gel (1 mg) was mixed with 200 mg KBr and pressed in a transparent pellet.

Scanning electron microscopy (SEM) using a microscope, Quanta 250 (equiped with EDAX detector, FEI Company, Eindhoven, The Netherlands) model was used in order to investigate the morphology of the gels. The preparation consisted of immobilizing on a double-sided carbon tape, with no coating. The EDAX determination shows the chemical composition at the surface.

### 4.3. Bacterial Strains, Media, and Growth Conditions

Two Gram-positive bacteria, *Staphylococcus aureus* ATCC 25923, *Virgibacillus halodenitrificans*, and Gram-negative bacterium *Escherichia coli* ATCC 25922, were used for antibacterial activity testing. Reference type strains were purchased from American Type Culture Collection (ATCC), Manassas, VA, USA. The halotolerant bacterium *Virgibacillus halodenitrificans* was isolated from a salt crystal from Unirea mine, Slănic Prahova, Romania. *E. coli* and *S. aureus* cells were cultured in Luria-Bertani (LB) broth at 37 °C with shaking at 180 rpm. *V. halodenitrificans* has grown on the MH culture medium, with the following composition (g/L): yeast extract, 10; proteose peptone 5; glucose 1; NaCl, 100; MgCl_2_ × 6H_2_0, 7; MgSO_4_ × 7H_2_O, 9.6; CaCl_2_ × 2H_2_O, 0.36; KCl 2; NaHCO_3_ 0.06; NaBr 0.026 [22,23] at 30 °C with shaking at 180 rpm. (All chemicals were purchased from Scharlab S.L., Sentmenat, Spain). The culture media pH was adjusted to 7.2 before sterilization.

### 4.4. Antibacterial Assay

The antimicrobial properties of gels treated at 400 °C, 3 h against bacterial strains was evaluated by the pour plate method. The overnight grown bacterial culture was diluted at an initial optical density of 0.15 at 660 nm (OD660 nm) and ready to be used for the antibacterial activity tests. The gel amounts used for the experiments were between 0.05 and 2 mg/mL. Each 10 mL of bacterial suspension was added in a tube test and mixed with different gels concentrations for the experimental procedure. The treated cells were incubated with shaking (150 rpm), for 24 h, at the temperature of 37 °C (*E. coli* and *S. aureus*), and for 48 h, at 30 °C (*V. halodenitrificans*). Aliquots of 1 mL were taken at defined intervals (4, 24 h for *E. coli* and *S. aureus* and 4, 24, 48 h for *V. halodenitrificans*), and serial dilutions were performed. Then, 100 µL of the appropriate dilutions were plated in duplicate on the culture medium. The Petri dishes were incubated at 37 °C or 30 °C, and the cell viability was assessed by enumerating the colony-forming unit per milliliter (CFU/mL) after 24 h. Untreated bacterial culture was served as a control with the cell viability of 100%. The number of colonies obtained was compared with the cells without treatment, and the survival percentage was calculated. The experiments were carried out in duplicate.

## Figures and Tables

**Table 1:** Main bands of gels in FTIR spectra and their assignations.

	Characteristic Bands of Gels in FTIR Spectra/cm^−1^								
S—100 °C	3435	1652	1209	1088	945	795	-	540	460
S—400 °C	3424	1633	1230	1104	-	800	-	-	460
G1—100 °C	3479	1652	1198	1082	943	793	575	-	456
1—400 °C	3435	1633	1216	1089	-	800	571	-	456
G2—100 °C	3460	1652	1198	1088	939	793	575	-	452
2—400 °C	3444	1633	1220	1084	-	800	571	-	452
Assignation	OH^–^	OH^–^	SiO_2_ Q3	Si_2_O_5_^2−^ Q2	Si_2_O_6_^4−^ Q1	SiO_4_^4−^ Q0	Zn–O–Zn	Si–O in rings	Si–O–Si

## Data Availability

The data presented in this study are contained within the article.
